# Enhanced Glutamate Synthesis and Export by the Thermotolerant Emerging Industrial Workhorse *Bacillus methanolicus* in Response to High Osmolarity

**DOI:** 10.3389/fmicb.2021.640980

**Published:** 2021-04-08

**Authors:** Christine Frank, Tamara Hoffmann, Oskar Zelder, Max F. Felle, Erhard Bremer

**Affiliations:** ^1^Laboratory for Microbiology, Department of Biology, Philipps-University Marburg, Marburg, Germany; ^2^Center for Synthetic Microbiology (SYNMIKRO), Philipps-University Marburg, Marburg, Germany; ^3^BASF SE, RWB/EC – A030 – L3/10, Ludwigshafen, Germany

**Keywords:** compatible solutes, stress responses, metabolism, secretion, biotechnology

## Abstract

The thermotolerant methylotroph *Bacillus methanolicus* MGA3 was originally isolated from freshwater marsh soil. Due to its ability to use methanol as sole carbon and energy source, *B. methanolicus* is increasingly explored as a cell factory for the production of amino acids, fine chemicals, and proteins of biotechnological interest. During high cell density fermentation in industrial settings with the membrane-permeable methanol as the feed, the excretion of low molecular weight products synthesized from it will increase the osmotic pressure of the medium. This in turn will impair cell growth and productivity of the overall biotechnological production process. With this in mind, we have analyzed the core of the physiological adjustment process of *B. methanolicus* MGA3 to sustained high osmolarity surroundings. Through growth assays, we found that *B. methanolicus* MGA3 possesses only a restricted ability to cope with sustained osmotic stress. This finding is consistent with the ecophysiological conditions in the habitat from which it was originally isolated. None of the externally provided compatible solutes and proline-containing peptides affording osmostress protection for *Bacillus subtilis* were able to stimulate growth of *B. methanolicus* MGA3 at high salinity. *B. methanolicus* MGA3 synthesized the moderately effective compatible solute L-glutamate in a pattern such that the cellular pool increased concomitantly with increases in the external osmolarity. Counterintuitively, a large portion of the newly synthesized L-glutamate was excreted. The expression of the genes (*gltAB* and *gltA2*) for two L-glutamate synthases were upregulated in response to high salinity along with that of the *gltC* regulatory gene. Such a regulatory pattern of the system(s) for L-glutamate synthesis in Bacilli is new. Our findings might thus be generally relevant to understand the production of the osmostress protectant L-glutamate by those Bacilli that exclusively rely on this compatible solute for their physiological adjustment to high osmolarity surroundings.

## Introduction

Several members of the genus *Bacillus* (e.g., *Bacillus subtilis*, *Bacillus licheniformis*, and *Bacillus megaterium*) are used as industrial cell factories for the manufacturing of bulk and fine chemicals and proteins of biotechnologically interest (Schallmey et al., 2004; Korneli et al., 2013; Van Dijl and Hecker, 2013; Su et al., 2020). There is an increasing demand for environmentally friendly and sustainable microbiologically based biotechnological processes that do not compete with the use of human or animal food-stocks. With a bio-economy in mind and the urgent need to reduce the climate-relevant CO_2_ foot-print of production processes, the C1-compound methanol receives rising attention, as methanol is a pure and readily available raw material that can be completely consumed by methylotrophic bacteria (Schrader et al., 2009; Puri, 2019). Hence, there is considerable interest in the biology and exploitation of these types of microorganisms for practical purposes (Lieven et al., 2018; Puri, 2019; Zhang et al., 2019).

*Bacillus methanolicus* (Schendel et al., 1990; Arfman et al., 1992; Heggeset et al., 2012) is a thermotolerant natural methylotroph and assimilates methanol via the ribulose monophosphate (RuMP) pathway (Müller et al., 2015b; Carnicer et al., 2016). It can grow at high temperature (optimally at 50°C) in minimal and rich media, and can use methanol as sole carbon and energy source. The natural ability of the *B. methanolicus* strain MGA3 (Naerdal et al., 2017) to synthesize and excrete large amounts (up to 59 g L^–1^ under fed-batch conditions) of the biotechnological important amino acid L-glutamate, makes this bacterium an interesting candidate to serve as an industrial cell factory for the conversion of the commodity chemical methanol into value-added products (Brautaset et al., 2007; Müller et al., 2015a).

During high-cell density fermentation in industrial settings, microbial cell factories experience various types of stresses that can negatively influence growth and the productivity of the entire biotechnological process (Schweder et al., 1999; Schweder, 2011; Nadal-Rey et al., 2021). Of particular note are osmotic challenges (Ronsch et al., 2003). These are caused through the addition of concentrated feed-solutions to the fermenter, and insufficient mixing of the feed will cause osmotic gradients in the fermenter broth (Borodina, 2019; Beitel et al., 2020; Betlej et al., 2020; Habicher et al., 2020; Yamakawa et al., 2020). The high-level accumulation of the desired low-molecular-weight compound(s) in the growth medium is also an issue when methanol is used as the feed. As methanol is membrane permeable, its addition to the fermenter will increase the overall osmolarity of the growth medium but it will not cause osmotic stress for the bacterial cell as the scale of the osmotic gradient difference across the cytoplasmic membrane is not altered (Wood, 2011; Bremer and Krämer, 2019). In contrast, an increase in the concentration of secreted membrane-impermeable low molecular weight compounds (e.g., L-glutamate) produced from methanol will have such an effect (Borodina, 2019).

In their varied ecological niches, most bacteria will experience osmotic stress and have to cope with it in a timely manner in order to keep cellular hydration, molecular crowding, and turgor within physiologically acceptable boundaries to sustain growth or to avoid cell rupture (Wood, 2011; Booth, 2014; Bremer and Krämer, 2019). Fluctuations in the external osmolarity disturb these processes as these will inevitably trigger water fluxes into (under hypoosmotic conditions) or out of the cell (under hyperosmotic conditions). As bacteria lack systems for an energy-dependent transport of water, the cell has to rely on indirect measures to counteract osmotically instigated changes in water fluxes across the semi-permeable cytoplasmic membrane (Wood, 2011; Booth, 2014; Bremer and Krämer, 2019). Many bacteria accomplish this at high osmolarity through the amassing, either via synthesis or import, of a selected group of highly water soluble low-molecular-weight organic osmolytes, the compatible solutes (Kempf and Bremer, 1998; Roeßler and Müller, 2001; Wood et al., 2001; Sleator and Hill, 2002). Compatible solutes are compliant with the biochemistry of the cell (Bolen and Baskakov, 2001; Yancey, 2005; Ignatova and Gierasch, 2006; Stadmiller et al., 2017). They can therefore be accumulated to exceedingly high intracellular pools to indirectly promote water retention and influx to maintain physiological adequate values of macromolecular crowding, hydration, and turgor under osmotically challenging conditions (Wood, 2011; Bremer and Krämer, 2019).

Members of the genus *Bacillus* make effective use of compatible solutes as protectants against high osmolarity induced cellular challenges (Hoffmann and Bremer, 2016). Depending on the species, Bacilli synthesize the compatible solutes L-glutamate, L-proline, or ectoine/hydroxyectoine (or a combination thereof) (Whatmore et al., 1990; Kuhlmann and Bremer, 2002; Bursy et al., 2007; Brill et al., 2011; Schroeter et al., 2013; Godard et al., 2020), and they can produce the osmostress protectant glycine betaine from prior imported choline (Boch et al., 1994, 1996). In addition, and as studied in detail in *B. subtilis*, several high-affinity uptake systems for compatible solutes operate in high-osmolarity stressed cells (Hoffmann and Bremer, 2017; Teichmann et al., 2018). *B. subtilis* can also generate osmostress-relieving L-proline pools from the import and metabolism of various amino acids and from the uptake and hydrolysis of proline-containing peptides (Zaprasis et al., 2013, 2015).

*Bacillus methanolicus* is emerging as a temperature-tolerant, methanol-based production host for value-added compounds (Brautaset et al., 2007; Müller et al., 2015a) and the synthesis of commercially interesting proteins (Irla et al., 2020). However, its ability to cope with osmotic stress is largely unexplored (Komives et al., 2005). It is thus of interest, both from the perspective of basic science and potential industrial uses of *B. methanolicus*, to understand the physiology of its adjustment processes to sustained high salinity/osmolarity surroundings. In our study, we discovered that in comparison with Bacilli already used as chassis for industrial-scale production processes (e.g., *B. subtilis*, *B. licheniformis*, and *B. megaterium*), *B. methanolicus* possesses a rather restricted ability to withstand sustained high osmolarity incurred stress. This property is probably linked to its exclusive synthesis of the moderately effective compatible solute L-glutamate, while *B. subtilis*, *B. licheniformis*, and *B. megaterium* all synthesize L-proline as their dominant compatible solute (Whatmore et al., 1990; Brill et al., 2011; Schroeter et al., 2013; Godard et al., 2020). Surprisingly, we observed that the *B. methanolicus* strain MGA3 cannot achieve osmostress resistance through import of compatible solutes or through the uptake and hydrolysis of proline-containing peptides, processes that make major contributions to the development of osmostress tolerance by *B. subtilis* (Hoffmann and Bremer, 2016, 2017).

## Results

### Growth of *B. methanolicus* MGA3 Under High-Salinity Conditions

**FIGURE 1:**
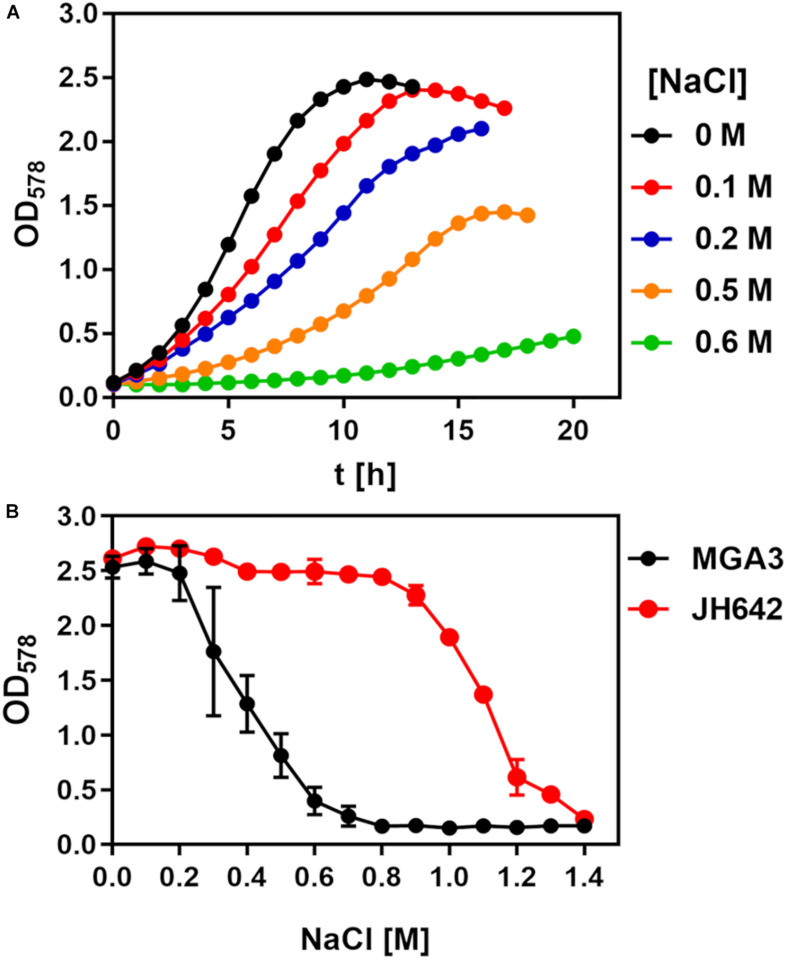
Growth of *Bacillus methanolicus* MGA3 challenged by high salinity. **(A)** *B. methanolicus* MGA3 was grown in MVcM in shake-flasks (20 mL culture volume in 100 mL Erlenmeyer flasks) at 50°C in the presence of the indicated NaCl concentrations. Growth of the cultures was monitored by measuring their OD_578_ values. The data shown represent a typical growth experiment. **(B)** Cultures of *B. subtilis* JH642 were grown in SMM at 37°C and *B. methanolicus* MGA3 was cultivated at 50°C in MVcM for 16 h, whereupon their growth yield was determined by measuring the OD_578_ of the cultures. The data shown were derived from two biological replicates.

To assess the salt tolerance of the *B. methanolicus* strain MGA3 (Schendel et al., 1990; Heggeset et al., 2012), we conducted two sets of growth experiments using a chemically defined minimal medium (MVcM) with methanol as the carbon and energy source and ammonium for the supply of nitrogen. In the first set of experiments, we systematically increased the salt concentration of the medium (from 0 M to 0.6 M NaCl) and monitored growth (at OD_578_) of the cultures in shake flasks. Accordingly, the osmolarity of the cultures was increased from 211 mOsmol kg^–1^ of the basal minimal medium MVcM to 1310 mOsmol kg^–1^ (MVcM with additional 0.6 M NaCl). Increases in the salinity of the medium concomitantly decreased the growth rate of *B. methanolicus* MGA3 from 0.29 h^–1^ when it was propagated in MVcM to 0.19 h^–1^ when the medium contained 0.5 M additional NaCl, conditions where a reasonable growth of the cultures still occurred (Figure 1A). However, growth of the cells in MVcM containing additional 0.6 M NaCl was severely impaired and resulted in a reduction of the growth rate to 0.08 h^–1^ (Figure 1A).

In the second set of experiments, we grew *B. methanolicus* MGA3 for a defined time (16 h) in MVcM with a broad range of salinities and then determined the growth yield of the cultures by measuring their OD_578_. We compared the growth profile of the salt-stressed *B. methanolicus* MGA3 cultures with that of the *B. subtilis* laboratory strain JH642 (Smith et al., 2014). This *B. subtilis* strain has previously been intensively studied with respect to its salt tolerance and the molecular mechanisms underlying this trait (Boch et al., 1994; Hoffmann and Bremer, 2016, 2017). *B. subtilis*, like other *Bacillus* species (Bursy et al., 2007; Schroeter et al., 2013; Godard et al., 2020), synthesizes large quantities of the compatible solute L-proline to counteract high salinity induced osmotic stress (Whatmore et al., 1990; Brill et al., 2011). In keeping with the data documented in Figure 1A, we found that *B. methanolicus* MGA3 is, in comparison with *B. subtilis* JH642, not particularly resistant to salt stress (Figure 1B).

### L-Glutamate Is the Only Compatible Solute Synthesized by *B. methanolicus* MGA3 in Response to High Osmolarity

**FIGURE 2:**
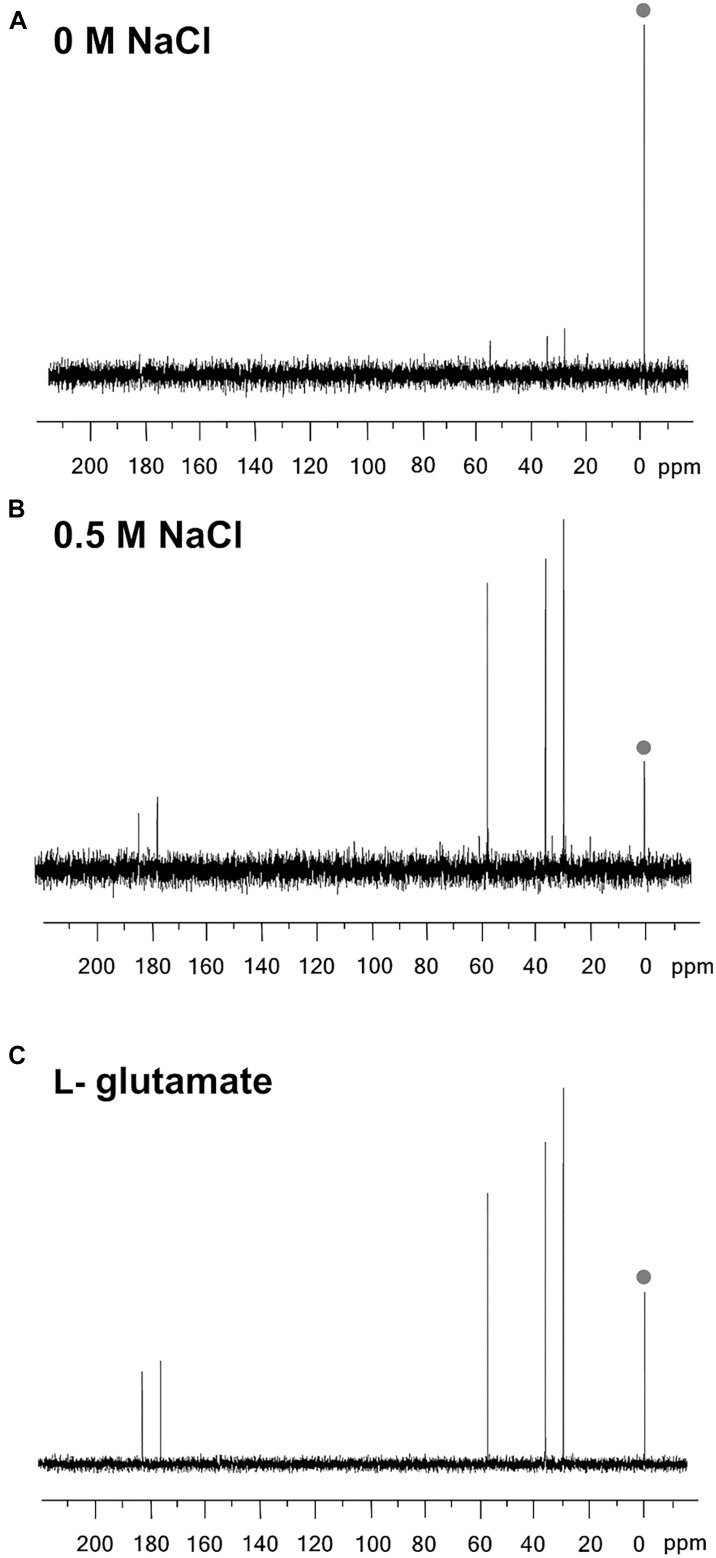
^13^C NMR spectra of ethanolic *Bacillus methanolicus* MGA3 cell extracts. Cultures of *B. methanolicus* MGA3 were grown at 50°C in MVcM in either **(A)** the absence, or **(B)** the presence of 0.5 M NaCl until they reached an OD_578_ of about 1. Ethanolic cell extracts were prepared and subjected to ^13^C-NMR analysis on a Bruker AC300 spectrometer operating at 75 MHz. D_4_-3-(trimethylsilyl)propionate was added to the extracts as an internal standard (its tracing is marked with a dot). **(C)** The ^13^C-NMR spectrum of L-glutamate (25 mg) was recorded as reference.

To identify the compatible solute(s) produced by *B. methanolicus* MGA3, we analyzed ethanolic cell extracts using natural abundance ^13^C-nuclear magnetic resonance spectroscopy (^13^C-NMR). ^13^C-NMR is a convenient and well-proven technique to identify compatible solutes synthesized by microorganisms under osmotic stress conditions (Kuhlmann and Bremer, 2002; Bursy et al., 2007). For this set of experiments, cultures of *B. methanolicus* MGA3 were grown to an OD_578_ of 1, either in MVcM without additional NaCl, or in MVcM containing 0.5 M NaCl. The ^13^C-NMR tracings revealed that *B. methanolicus* MGA3 produces exclusively the compatible solute L-glutamate in response to sustained osmotic stress (Figure 2).

Some microorganisms can switch the synthesis from one particular compatible solute to another, either in response to changes in growth phase or further increases in salinity (Saum and Müller, 2007; Kuhlmann et al., 2008). We therefore also assessed the compatible solute profile of *B. methanolicus* MGA3 cells in cultures grown to stationary phase in the absence (MVcM) or in the presence of increased salinity (MVcM with 0.5 M NaCl). Again, L-glutamate was the only compatible solute synthesized by salt-stressed stationary phase *B. methanolicus* MGA3 cells (Supplementary Figure 1). This is consistent with our *in silico* analysis of the *B. methanolicus* MGA3 genome sequence (Heggeset et al., 2012), as we did not find genes for osmostress adaptive synthesis of L-proline (Brill et al., 2011; Schroeter et al., 2013; Godard et al., 2020), ectoine/hydroxyectoine (Czech et al., 2018), trehalose (Wolf et al., 2003), or for the choline-dependent synthesis of glycine betaine (Boch et al., 1996).

In the above described studies (Figures 1A,B), we used NaCl to impose osmotic stress onto *B. methanolicus* MGA3 cells. We therefore wondered whether the observed enhanced production of L-glutamate was a salt-stress specific effect, or whether it was actually trigged by high osmolarity. Consequently, we monitored L-glutamate production by *B. methanolicus* MGA3 cells via high-performance liquid chromatography (HPLC) in cultures that were osmotically challenged either with ionic (NaCl and KCl), or non-ionic (sucrose and lactose) solutes. These solutes were added to the cultures in quantities such that the cells were exposed to approximately the same degree of osmolarity. Both ionic and non-ionic solutes triggered the formation of an approximately three-fold increased intracellular L-glutamate pool (Table 1). Hence, increased synthesis of the compatible solute L-glutamate is a cellular response of *B. methanolicus* MGA3 to a true osmotic challenge.

**TABLE 1:** Intracellular L-glutamate content of *Bacillus methanolicus* MGA3 and of supernatants of cells grown in MVcM supplemented with either ionic or non-ionic osmolytes.

Medium	Osmolarity [mOsmol kg^–1^]	Intracellular glutamate [mg g(CDW)^–1^]	Extracellular glutamate [mg g(CDW)^–1^]
MVcM	211	16 ± 5	19 ± 6
MVcM 0.5 M NaCl	1056	51 ± 15	186 ± 75
MVcM 0.5 M KCL	1105	43 ± 9	190 ± 46
MVcM 0.64 M sucrose	1125	49 ± 3	93 ± 27
MVcM 0.65 M lactose	1130	46 ± 2	77 ± 13

*Cultures of B. methanolicus MGA3 were grown in MVcM in the absence or presence of the indicated ionic and non-ionic osmolytes to an OD_578_ of about 1 using 200 mM methanol as the sole carbon and energy source. The osmolarity of the growth media were determined with a freezing point osmometer and L-glutamate was quantitated by HPLC analysis. The data shown were derived from two biological replicates and each sample was assayed twice.*

*B. methanolicus* strain MGA3 is known to excrete large amounts of L-glutamate under fed-batch conditions, or when it is cultivated under Mg^2+^ limitations in shake flasks (Schendel et al., 1990; Krog et al., 2013; Irla et al., 2017). The physiological and genetic reason(s) why export of L-glutamate in this particular isolate occurs under these special growth conditions is unknown (Heggeset et al., 2012). Although counterintuitive for the functioning of compatible solutes in conferring osmostress tolerance (Wood, 2011; Bremer and Krämer, 2019), we wondered whether *B. methanolicus* strain MGA3 would also excrete substantial amounts of L-glutamate under osmotic stress conditions. Accordingly, we monitored the L-glutamate content of the supernatants of cultures of osmotically stressed cells via HPLC analysis (Table 1). An approximately nine-fold increase in L-glutamate content of the medium was found in cultures grown in the presence of 0.5 M NaCl or 0.5 M KCl (Table 1). An increased L-glutamate content was also detected in the supernatant of cells osmotically challenged with non-ionic solutes sucrose and lactose. However, for unknown reasons, the external L-glutamate pool was lower in these cultures in comparison with those exposed to ionic osmolytes; there was about only a four-fold increase in L-glutamate content compared with osmotically non-stressed cells (Table 1). This difference cannot be caused by the metabolism of the used sugars, as these, in contrast to glucose and mannitol, are not catabolized by *B. methanolicus* MGA3 (Schendel et al., 1990; Supplementary Figure 2).

### *B. methanolicus* MGA3 Secretes Most of the Newly Synthesized L-Glutamate Under Osmotic Stress Conditions

**FIGURE 3:**
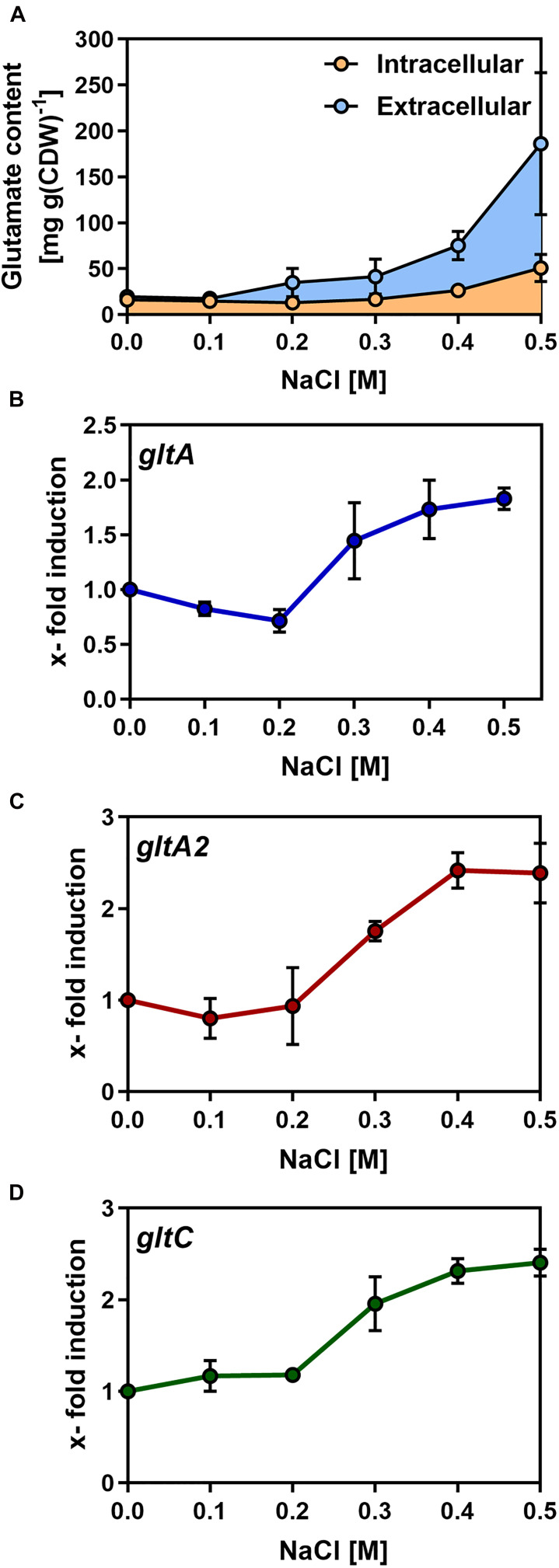
Synthesis of L-glutamate by *Bacillus methanolicus* MGA3 by osmotically stressed *B. methanolicus* cells. **(A)** Cultures of *B. methanolicus* MGA3 were grown at 50°C in MVcM containing the indicated NaCl concentration to an OD_578_ of approximately 1. Intracellular and extracellular L-glutamate was quantified by HPLC analysis. The shown data represent experiments from three biological replicates. **(B,C)** qPCR analysis of L-glutamate synthase encoding genes (*gltA* and *gltA2*) and of the *gltC* regulatory gene of *B. methanolicus* MGA3. qPCR data for **(B)** *gltA*, **(C)** *gltA2*, and **(D)** *gltC* were derived from cultures of *B. methanolicus* MGA3 grown at 50°C in MVcM containing the indicated concentrations of NaCl. The relative quantification of transcript levels is given; all values were compared to the values of the *gltA*, *gltA2*, and *gltC* transcripts detected in *B. methanolicus* MGA3 cells grown in MVcM in the absence of additional NaCl; the cellular levels of these mRNAs was set at one. For internal normalization of mRNAs, the sequence of the *B. methanolicus* MGA3 16S rRNA was used. The presented data were derived from RNA preparations of two independently grown *B. methanolicus* MGA3 cultures and each RNA preparation was measured three-times.

Having shown that *B. methanolicus* MGA3 secretes L-glutamate also under osmotic stress conditions (Table 1), we analyzed the intracellular and extracellular L-glutamate pools formed by *B. methanolicus* MGA3 in greater detail by HPLC analysis. For these experiments, we propagated *B. methanolicus* MGA3 cultures under conditions with systematically increased levels of salinity. Concomitant with the increase in salinity of the growth medium, enhanced pools of L-glutamate were produced by the cells (Figure 3A). Strikingly, most of the L-glutamate synthesized under osmotically stress conditions was excreted, and this phenomenon was in particular notably at higher salinities. For instance, *B. methanolicus* MGA3 cells grown in MVcM containing additional 0.5 M NaCl, secreted about 70% of all newly synthesized L-glutamate into the medium (Figure 3A). Overall, the combined intracellular and extracellular L-glutamate pools increased from 36 mg L-glutamate g dry weight^–1^ in cultures grown in MVcM to 220 mg L-glutamate g dry weight^–1^ in cultures propagated in MVcM containing 0.5 M additional NaCl. Hence, the overall production of this compatible solute increased about six-fold upon the imposition of sustained osmotic stress (Figure 3A). When only the extracellular L-glutamate content is considered, cultures of *B. methanolicus* MGA3 grown in MVcM containing 0.5 M NaCl produced 80 mg L^–1^ of this amino acid when the cells were grown at 50°C in shake flasks.

### Expression of L-Glutamate Synthase Encoding Genes Are Upregulated in Response to High Osmolarity

**FIGURE 4:**
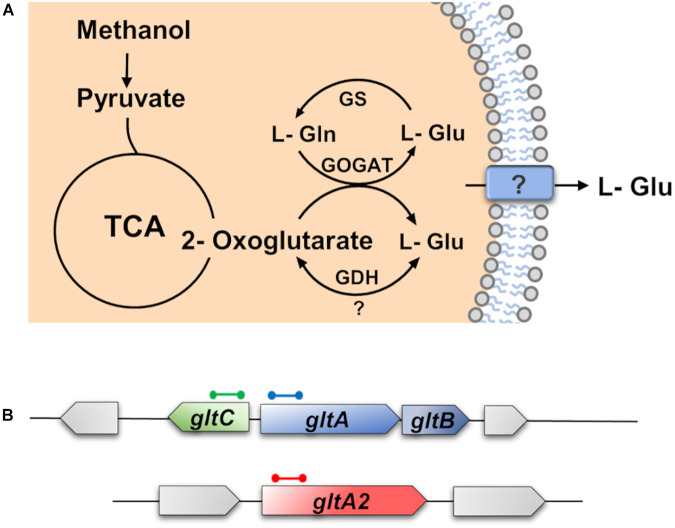
Schematic overview of the synthesis and degradation of L-glutamate by *Bacillus methanolicus* MGA3. **(A)** The 2-oxoglutarate and L-glutamine are converted to L-glutamate by the L-glutamate synthase (GOGAT) [via the GltAB and GltA2 enzymes (Krog et al., 2013)] and most of it is excreted by *B. methanolicus* MGA3 through an unknown transporter, or channel protein. In addition, L-glutamate can be synthesized by the glutamate dehydrogenase (GDH) or can be catabolically degraded by the GDH (Krog et al., 2013). This reaction is dependent on the affinity of the GDH to ammonia, 2-oxoglutarate, and L-glutamate. L-glutamate can also be degraded by the glutamine synthetase (GS) to L-glutamine. **(B)** Genetic organization of the L-glutamate synthesis genes (GOGAT) encoded by the *gltAB* operon and of *gltA2*, and of the regulatory gene *gltC*. The genetic organization of the *gltAB*, *gltC*, and *gltA2* structural genes and their genome neighborhood was deduced from the *B. methanolicus* MGA3 genome sequence (Heggeset et al., 2012).

The biochemistry of L-glutamate synthesis in *B. methanolicus* MGA3 has previously been studied both *in vitro* and *in vivo* (Krog et al., 2013). As reported by Krog et al. (2013), *B. methanolicus* MGA3 has two active glutamate synthases (GltAB and GltA2). The GltAB enzymes form a complex [large subunit (GltA) and small subunit (GltB), respectively], while the GltA2 enzyme seems to operate without the small GltB subunit (Krog et al., 2013). It possesses also a glutamate dehydrogenase (GDH) (YweB) (Figure 4A). In contrast to *B. subtilis* (Commichau et al., 2007b; Stannek et al., 2015; Dormeyer et al., 2019), the major function of the GDH of *B. methanolicus* MGA3 seems to be centered on L-glutamate synthesis, rather than its degradation as judged by the kinetic parameters of the GDH: K_*m*_ (L-Glu) = 250 mM; K_*m*_ (ammonium) = 10 mM; K_*m*_ (2-oxoglutarate) = 20 mM). The corresponding *V*_*max*_ values of this enzyme are 10 U mg^–1^ for L-glutamate synthesis; and 1.4 U mg^–1^ for L-glutamate degradation (Krog et al., 2013).

The *gltAB* genes co-localize with their presumed *gltC* regulatory gene on the *B. methanolicus* MGA3 genome, while the *gltA2* gene is positioned elsewhere on the chromosome (Figure 4B); GltA2 possesses an amino acid sequence identity with GltA of 29% (Heggeset et al., 2012). It is unknown whether GltC plays any role in controlling *gltA2* transcription. Extensive studies with *B. subtilis* revealed that the LysR-type regulatory protein GltC functions both as an activator and as a repressor for *gltAB* transcription along with other transcription factors and the moonlighting L-glutamate dehydrogenase (GDH) enzymes (RocG and GudB) in a rather complex sequence of events (Sonenshein, 2007; Gunka and Commichau, 2012; Dormeyer et al., 2019). No corresponding genetic or biochemical data are available for the *gltAB* and *gltA2* genes of *B. methanolicus* MGA3.

Given that enhanced L-glutamate production in *B. methanolicus* MGA3 is triggered by high osmolarity (Figure 3A), we considered the possibility that the transcription of the genes encoding the two GOGAT enzymes (*gltAB* and *gltA2*), their putative regulatory gene *gltC* (Figure 4B) and the gene (*yweB*) for the GDH enzyme were upregulated in response to sustained osmotic stress. We therefore monitored the expression of these genes by quantitative PCR (qPCR), setting the basal levels of *gltA*, *gltA2*, and *gltC* transcription in cell grown in MVcM as one. These three genes exhibited a very similar pattern of transcription under increased osmotic stress conditions: up to the addition of 0.2 M NaCl to MVcM, no significant increase in expression levels were recorded, while further increases in the salinity of the growth medium triggered successively enhanced levels of expression (Figures 3B–D). Using the same mRNA preparations employed to study the osmotically induced transcription of the *gltAB, gltA2*, and *gltC* genes, we found that the transcriptional profile of *yweB* was not increased in response to high salinity (Supplementary Figure 2).

### Osmostress Experienced by *B. methanolicus* MGA3 Cannot Be Relieved by an Exogenous Supply of a Broad Range of Compatible Solutes

**FIGURE 5:**
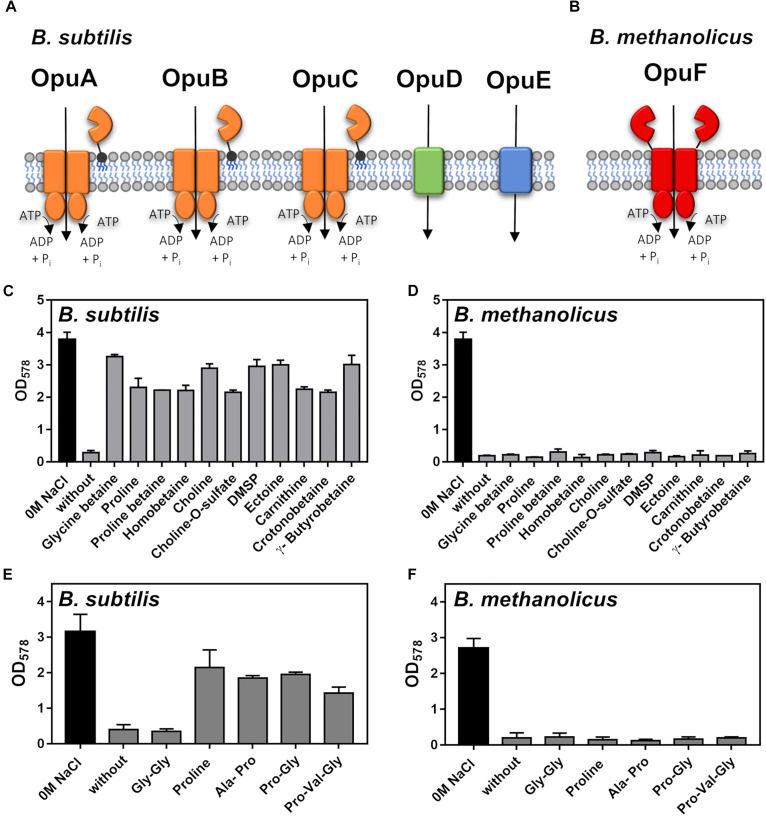
Osmostress protection of *Bacillus subtilis* JH642 and *Bacillus methanolicus* MGA3 by externally provided compatible solutes and proline-containing peptides. **(A)** Schematic representation of the five Opu compatible solute transporters operating in *B. subtilis* (Hoffmann and Bremer, 2017) and **(B)** of the *B. methanolicus* MGA3 OpuF-type ABC transporter as deduced from the genome sequence (Heggeset et al., 2012) and previous studies of this transporter family (Teichmann et al., 2018). **(C)** Cultures of *B. subtilis* JH642 were grown either in SMM or in SMM with a growth-inhibiting concentration of NaCl (1.2 M) in the absence or the presence of 1 mM of the indicated osmostress protectants. Growth of the cultures was monitored by measuring their OD_578_ after 18 h of incubation at 37°C in a shaking water bath. The data shown were derived from two biological replicates. **(D)** Growth of *B. methanolicus* MGA3 at 50°C either in MVcM or in MVcM with a growth-inhibiting concentration of NaCl (0.6 M) in the absence or in the presence of 1 mM of the indicated osmostress protectants. The data shown were derived from two biological replicates. **(E,F)** Growth of **(E)** the *B. subtilis* strain GWB100 (at 37°C in SMM containing 1.2 M NaCl) and **(F)** *B. methanolicus* MGA3 [at 50°C in either in MVcM or in MVcM with a growth-inhibiting concentration of NaCl (0.6 M)] in the presence of 1 mM of L-proline or the L-proline-containing the indicated peptides. Growth of the cultures was monitored by measuring their OD_578_ after 18 h of incubation. The data shown were derived from two biological replicates.

In addition to the synthesis of compatible solutes, many bacteria can relieve the negative consequences of high osmolarity on cellular physiology and growth through osmotically stimulated import of compatible solutes (Kempf and Bremer, 1998; Roeßler and Müller, 2001; Wood et al., 2001; Sleator and Hill, 2002). This process has been intensively studied in *B. subtilis* (Hoffmann and Bremer, 2016), where the activities of five osmoprotectant uptake systems (Opu) (Figure 5A) with different substrate profiles provide cellular protection (Hoffmann and Bremer, 2017). Opu-type import systems for compatible solutes are widely found in members of the genus *Bacillus* (Teichmann et al., 2018). However, we observed that no genes for any of the five Opu-type transporters operating in *B. subtilis* are present in the genome sequence of *B. methanolicus* MGA3 (Heggeset et al., 2012).

In contrast to *B. subtilis*, *B. methanolicus* MGA3 possesses genes (*opuFA-opuFBC*) (BMMGA3_01135 - BMMGA3_01130) for an OpuF-type ABC transporter (Teichmann et al., 2018; Figure 5B). While the substrate binding proteins of the *B. subtilis* OpuA, OpuB and OpuC transporters are tethered via a lipid modification of their mature N-termini to the outer face of the cytoplasmic membrane (Figure 5A; Hoffmann and Bremer, 2016, 2017), the extracellular substrate binding domain of OpuF-type ABC transporters is fused to the transmembrane domain (Teichmann et al., 2018). The OpuF systems of *Bacillus infantis* and *Bacillus panaciterrae* serve for the uptake of a restricted number of compatible solutes; e.g., glycine betaine, proline betaine, homobetaine, and DMSP (Teichmann et al., 2018; Sikkema et al., 2020). However, it is worth noting that OpuF-related systems exists that are seemingly not involved in compatible solute import (Ruiz et al., 2016; Herrou et al., 2017).

The predicted overall fold for the extracellular OpuFC substrate binding domain (Supplementary Figure 4A) resembles that of many other substrate binding proteins operating in conjunction with bacterial ABC transporters (Berntsson et al., 2010). It possesses an aromatic ligand-binding-cage (Supplementary Figure 4B) that can accommodate fully methylated head-groups of compatible solutes (e.g., glycine betaine, arsenobetaine, carnitine, choline, proline betaine, and DMSP) via cation-pi interactions (Hoffmann and Bremer, 2017; Teichmann et al., 2018; Bremer and Krämer, 2019).

Despite the presence of an OpuF-type ABC transporter system in *B. methanolicus*, none of the 11 externally provided compatible solutes providing osmostress protection to *B. subtilis* (Figure 5C) was able to afford cellular protection by promoting growth of *B. methanolicus* MGA3 at high salinity (Figure 5D). The genes encoding the *B. methanolicus* MGA3 OpuF system are not transcriptionally upregulated by high salinity (Supplementary Figure 4C) and the genome sequence of the *B. methanolicus* strain PB1 (Heggeset et al., 2012), an isolate originally retrieved from the wastewater treatment system of a sugar beet factory (Arfman et al., 1992; Komives et al., 2005), lacks not only all Opu systems present in *B. subtilis*, but OpuF is absent as well.

### *B. methanolicus* MGA3 Cannot Derive Osmostress Protection From Proline-Containing Peptides

L-proline serves as a major compatible solute for both bacteria and plants (Fichman et al., 2014). *B. subtilis* is such a L-proline producer and L-proline synthesis is a crucial physiological determinant for its ability to withstand high osmolarity induced cellular stress (Whatmore et al., 1990; Brill et al., 2011). Osmostress protection of *B. subtilis* can also be accomplished through import of L-proline via the osmotically controlled OpuE transporter (Von Blohn et al., 1997), or the import and intracellular hydrolysis of proline-containing peptides (Zaprasis et al., 2013). Proline-containing peptides are present in components of rich media (e.g., yeast extract and peptone) and many bacteria often possess different types of peptide uptake systems. Genes for three peptide transporters (Dpp, Opp, and DtpT) are present in *B. methanolicus* MGA3 (Heggeset et al., 2012), and this strain also possesses orthologs of those *B. subtilis* peptidases (PapA and PapB) that liberate L-proline from imported peptides for osmostress protective purposes (Zaprasis et al., 2013). When both L-proline and three L-proline containing peptides (Ala-Pro, Pro-Val-Gly, and Pro-Gly) were tested that can provide osmostress protection to *B. subtilis* (Zaprasis et al., 2013; Figure 5E), none stimulated growth of *B. methanolicus* MGA3 at high osmolarity (Figure 5F).

### *B. methanolicus* MGA3 Cannot Use L-Glutamate as Nitrogen or Sole Carbon and Energy Source

**FIGURE 6:**
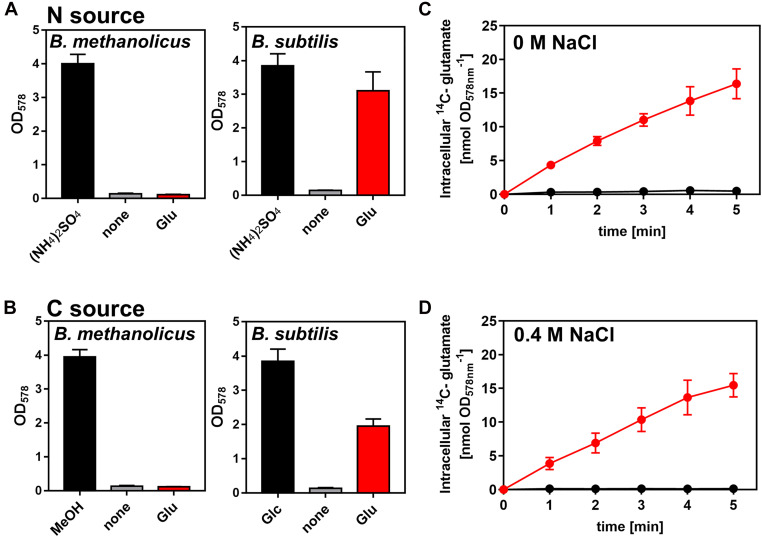
*Bacillus methanolicus* MGA3 is unable to use externally provided L-glutamate as a nutrient. **(A,B)** Use of L-glutamate by *B. methanolicus* strain MAG3 and by *B. subtilis* strain H642 either as **(A)** sole nitrogen (N) or **(B)** as sole carbon (C) source. **(A)** 16 mM (NH_4_)_2_SO_4_ present in the SMM and MVcM minimal media was replaced by 32 mM L-glutamate for the cultivation of *B. methanolicus* MGA3 and *B. subtilis* JH642 when the use of L-glutamate was assessed as nitrogen source. **(B)** When the use of L-glutamate as sole carbon and energy source was tested, it was provided at a concentration of 40 mM. The reference cultures were grown either in the presence of 200 mM methanol (for *B. methanolicus* MGA3 at 50°C) or 28 mM glucose (for *B. subtilis* JH642 at 37°C). Growth yield of the cultures was determined after 18 h of incubation by determining the OD_578_ value. All data shown were derived from two biological replicates. **(C,D)** The uptake of radiolabeled L-[U-^14^C]glutamate by *B. methanolicus* MGA3 (black) and *B. subtilis* JH642 (red) at a final concentration of 1 mM was measured either the absence **(C)** or presence **(D)** of 0.4 M NaCl in the used culture media (SMM for *B. subtilis* JH642 and MVcM for *B. methanolicus* MGA3, respectively). The data shown were derived from two biological replicates.

L-glutamine is a preferred nitrogen source for *B. subtilis* as its synthesis requires only one molecule of ATP (Sonenshein, 2007; Gunka and Commichau, 2012; Dormeyer et al., 2019). Only *B. subtilis* strains with functional and catabolically active GDHs can grow with L-glutamate as sole nitrogen and carbon source (Belitsky and Sonenshein, 1998; Commichau et al., 2008). Since *B. methanolicus* MGA3 produces copious amounts of L-glutamate (Brautaset et al., 2003) and secretes most of it under osmotic stress conditions (Figure 3A), we wondered if this amino acid could be re-imported and used as a nutrient. In the genome sequence of *B. methanolicus* MGA3 a gene (*gltT*) (BMMGA3_08695) is present encoding a sodium-driven L-glutamate importer (GltT), a system that serves for the high-affinity uptake of L-glutamate and L-aspartate in *B. subtilis* (Zaprasis et al., 2015). However, our growth data showed that *B. methanolicus* MGA3 cannot use L-glutamate as a nutrient, neither as sole nitrogen nor as sole carbon source (Figures 6A,B). Consistent with these physiological data is our observation that *B. methanolicus* MGA3 cannot import L-glutamate (at least not under the tested conditions), while osmotically stressed or non-stressed *B. subtilis* cells acquire this amino acid from an external source (Figures 5C,D). The inability of *B. methanolicus* MGA3 to use L-glutamate as a nutrient is in all likelihood a reflection by the above outline characteristics of its sole GDH (YweB) enzyme (Krog et al., 2013).

## Discussion

The thermophilic Gram-positive methylotroph *B. methanolicus* MGA3 is increasingly recognized as a potential industrial workhorse (Brautaset et al., 2007; Müller et al., 2015a) for the commodity production of various amino acids, fine chemicals, and recombinant proteins using the readily replenishable and non-food C1 feed-stock methanol (Brautaset et al., 2003, 2010; Naerdal et al., 2015, 2017; Irla et al., 2016, 2020; Hakvag et al., 2020). Keeping in mind that industrial fed-batch production processes will expose the producer microorganisms to high osmolarity surroundings (Ronsch et al., 2003; Zou et al., 2016; Borodina, 2019; Beitel et al., 2020; Habicher et al., 2020; Yamakawa et al., 2020), we studied how *B. methanolicus* MGA3 copes physiologically with this challenge and how this constraint on growth could potentially be alleviated.

In line with the ecophysiological circumstances in fresh-water marsh soils from which *B. methanolicus* MGA3 was originally isolated (Schendel et al., 1990), we found that it is a rather salt-sensitive member of the Bacilli (Kuhlmann and Bremer, 2002; Bursy et al., 2007). Furthermore, it seems to lack import systems for compatible solutes. This precludes the simple amelioration of high osmolarity triggered cellular stress through the addition of commercially readily available osmostress protectants (e.g., glycine betaine) to the fermentation medium (Ronsch et al., 2003; Zou et al., 2016). *B. methanolicus* MGA3 can also not take advantage of L-proline-containing peptides that are present in yeast extract, peptone and other components of rich media, compounds that are imported and hydrolyzed by *B. subtilis* in order to replenish its osmostress protective L-proline pool (Zaprasis et al., 2013). Hence, so far, no system could be identified in *B. methanolicus* MGA3 that would offset its osmotic sensitivity through the import of low-molecular-weight compounds. Given that osmostress protectant uptake systems are widely present in members of the genus *Bacillus* (Hoffmann and Bremer, 2017; Teichmann et al., 2018), this is a rather surprising finding.

The degree of osmotolerance exhibited by Bacilli is linked to the type(s) of compatible solute(s) that they produce. Although certainly not the only determinant to achieve this trait (Hoffmann and Bremer, 2016), stress resistance seems to increase in this group of microorganisms according to the type of the synthesized compatible solute in the following order: L-glutamate – L-proline – ectoine/hydroxyectoine (Kuhlmann and Bremer, 2002; Bursy et al., 2007). Consistent with this scheme, we found that the rater osmotically sensitive *B. methanolicus* strain MGA3 produces exclusively L-glutamate in response to sustained osmotic stress. Like other compatible solutes (Ignatova and Gierasch, 2006; Street et al., 2006; Stadmiller et al., 2017), L-glutamate not only serves as an osmostress protectant but also functions as a chemical chaperone (Diamant et al., 2001; Yancey, 2005) by promoting the proper folding and functionality of proteins and other cell components (Leirmo et al., 1987; Deredge et al., 2010; Cheng et al., 2016; Kozlov et al., 2017; Purohit et al., 2021). A pattern of osmotically induced production of L-glutamate similar to that reported here for *B. methanolicus* MGA3 has formerly also been observed for the formidable food pathogen *Bacillus cereus*, a rather salt-sensitive member of the Bacilli as well (Kuhlmann and Bremer, 2002).

While previously not studied for *B. cereus*, we found that the transcriptional profile of the *B. methanolicus* MGA3 *gltA*, *gltA2* and *gltC* genes mirrors the profile of L-glutamate production under osmotic stress conditions (Figure 3). Hence, osmotically stimulated L-glutamate production must be, at least in part, be dependent on enhanced transcription of the genes encoding the two GOGAT enzymes operating in *B. methanolicus* MGA3 and of the correlated *gltC* regulatory gene. Our findings thus imply that the paradigm for the genetic and biochemical control of L-glutamate synthesis and catabolism that emerged from in depth studies focusing on *B. subtilis* [for an excellent recent overview (see Dormeyer et al., 2019)] cannot completely hold for the corresponding process in *B. methanolicus*.

In *B. subtilis*, high cellular L-glutamate pools, along with a catabolically active moonlighting GDH enzyme (RocG or GudB), are required for the GltC regulator to repress transcription of the L-glutamate biosynthetic *gltAB* operon (Belitsky and Sonenshein, 2004; Commichau et al., 2007a; Stannek et al., 2015; Dormeyer et al., 2019). Such a repression of gene transcription of L-glutamate biosynthetic genes needs to be avoided by those Bacilli that use L-glutamate as their dominant osmostress protectant (Kuhlmann and Bremer, 2002; Bursy et al., 2007). In this process, the L-glutamate pool needs to increase to high cellular levels. Hence, it is apparent that central elements of the complex genetic and biochemical control of L-glutamate pools in *B. subtilis* must be substantially different in those Bacilli that use L-glutamate as their dominant compatible solute. An important element seems to be the absence of a catabolic GDH in *B. methanolicus* MGA3 (Krog et al., 2013), while in *B. subtilis* enzymatically active GHDs (RocG and GudB) act as co-regulators of the transcription factor GltC to ultimately control glutamate synthase activity (Belitsky and Sonenshein, 1998; Commichau et al., 2007a; Dormeyer et al., 2019).

*B. methanolicus* MGA3 is unusual as it naturally secretes large quantities of L-glutamate when it is cultivated under fed-batch conditions in fermenters, or exposed to Mg^2+^ limitation (Brautaset et al., 2007, 2010; Irla et al., 2017). The underlying physiological purpose and genetic determinants of this process are unclear. No clues regarding these issues could be deduced from an analysis of the *B. methanolicus* MGA3 genome sequence in comparison with that of the *B. methanolicus* strain PB1. This isolate was originally retrieved from the wastewater treatment system of a sugar beet factory (Arfman et al., 1992; Komives et al., 2005), and in contrast to strain MGA3 does not secrete large amounts of L-glutamate (Heggeset et al., 2012).

In order for compatible solutes to function as osmostress protectants, the cell ties their synthesis and intracellular accumulation to the degree of the imposed osmotic stress. This allows it a finely tuned physiological adjustment to the prevailing osmotic conditions in its surroundings (Kempf and Bremer, 1998; Roeßler and Müller, 2001; Wood et al., 2001; Sleator and Hill, 2002). This pattern is also observed for the production of L-glutamate by *B. methanolicus* MGA3 (Figure 3A). However, it is extraordinary that even under osmotic stress conditions, a large portion of the newly synthesized L-glutamate is secreted, where it cannot contribute to the cellular osmostress adjustment process (Figure 3A). This contradictory and seemingly wasteful behavior of strain MGA3 is not easily reconciled with the role of L-glutamate as an osmostress protectant. Perhaps, the secretion of L-glutamate under osmotic stress conditions is a peculiarity of this particular strain because *B. methanolicus* MGA3 already secretes large amounts of this amino acid under special growth conditions (Brautaset et al., 2007, 2010; Irla et al., 2017). However, it should also be noted that microorganisms seem to excrete compatible solutes even under sustained high osmolarity steady-state conditions and subsequently re-import them again (Lamark et al., 1992; Grammann et al., 2002; Hoffmann et al., 2012). This *synthesis-release-recapture* cycle might serve to aid osmotically stressed cells to fine-tune their turgor when they double their volume prior to division (Hoffmann et al., 2012). Notably, when the uptake system for the particular compatible solute synthesized by a given bacterium is non-functional, compatible solutes will accumulate in the growth medium and thereby limiting the full potential of the cell’s osmostress adjustment systems (Hoffmann et al., 2012). This is exactly what might happen with *B. methanolicus* strain MGA3 as it cannot import L-glutamate under either low or high salinity growth conditions (Figures 6C,D).

Many microorganisms possess exporters for amino acids, systems that become highly relevant when bacterial cell factories are used for industrial scale production of these commercially interesting compounds (Krämer, 1994; Eggeling and Sahm, 2003; Borodina, 2019). To the best of our knowledge, export systems for L-glutamate have only been identified at the molecular level in *Corynebacterium glutamicum*, the premier industrial production host for L-glutamate (Wendisch, 2020). Surprisingly, these export systems are members of the MscS family of mechanosensitive channels (Kawasaki and Martinac, 2020; Nakayama, 2021). These types of channels are typically used as tightly controlled safety-valves in bacteria to withstand osmotic down-shocks (Booth, 2014). The main L-glutamate exporter of *C. glutamicum* is the MscCG channel. Its monomer exhibits a *trans-*membrane topology deviating from that of archetypical MscS-type mechanosensitive channels and can thus readily be identified via bioinformatics (Kawasaki and Martinac, 2020; Nakayama, 2021). We found that an MscCG-type channel is not encoded by the *B. methanolicus* MGA3 genome (Heggeset et al., 2012), and the question whether the present traditional MscS-type channel is involved in L-glutamate secretion is unanswered.

Given that L-glutamate excretion by *B. methanolicus* MGA3 can be triggered not only by high osmolarity (Figure 3A), but also by manipulating its growth conditions (Schendel et al., 1990; Krog et al., 2013; Irla et al., 2017), this thermotolerant methylotroph might be a useful system to further explore the molecular determinants of L-glutamate export in bacteria. Furthermore, the physiological and genetic results presented here might serve as a primer to understand osmotic adjustment in the considerable group of those Bacilli that rely on the production of L-glutamate as their sole compatible solute (Kuhlmann and Bremer, 2002; Bursy et al., 2007).

## Materials and Methods

### Bacterial Strains

The *B. methanolicus* MGA3 strain (Schendel et al., 1990; Heggeset et al., 2012) was provided for studies conducted at the Philipps-University Marburg by BASF SE (BASF SE, Ludwigshafen, Germany). The *B. subtilis* strain JH642 (*trpC2 pheA1*; BGSC 1A96) is a derivative of the *B. subtilis* domesticated wild-type laboratory strain 168 (Smith et al., 2014). The *B. subtilis* strain GWB100 is an *appA*^+^ derivative of *B. subtilis* JH642 that is defective [Δ(*proHJ:tet*)] for osmostress adaptive L-proline biosynthesis and is therefore osmotically sensitive (Brill et al., 2011). It was used as host to assess the use of proline-containing peptides as osmostress protectants (Zaprasis et al., 2013).

### Chemicals, Media, and Growth Conditions

All compatible solutes used in this study were from laboratory stocks as described previously (Hoffmann and Bremer, 2011). Radiolabeled L-[U-^14^C]glutamate (253 mCi mmol^–1^) was purchased from GE Healthcare Lifesciences (Munich, Germany). ^2^H_2_O was obtained from Aldrich (Deisenhofen, Germany) and D_4_-3-(trimethylsilyl)propionate (Fisher Scientific GmbH; Deisenhofen, Germany) dissolved in ^2^H_2_O was used as internal standard in ^13^C-NMR experiments. For high-performance liquid chromatography (HPLC) analysis, L-glutamate, and the derivatization reagents for amino acids, *o*-phtaldialdehyde (OPA) and fluorenylmethyloxycarbonyl chloride (FMOC-Cl), were purchased from Sigma-Aldrich (St. Louis, United States). Acetonitrile (HPLC grade) was obtained from VWR International GmbH (Heidelberg, Germany). Methanol was purchased from Carl Roth GmbH (Karlsruhe, Germany) and was of HPLC grade.

The *B. subtilis* strains JH642 and GWB100 were routinely maintained and propagated on LB agar plates, or cultured in LB liquid medium at 37°C. *B. methanolicus* MGA3 was propagated on SOB agar plates or cultured in SOB liquid medium at 50°C (Hanahan, 1983). Spizizen’s minimal medium (SMM) with 0.5% glucose as a carbon source, a solution of trace elements (Harwood and Archibald, 1990), and L-tryptophan (20 mg L^–1^) and L-phenylalanine (18 mg L^–1^) was used as a chemically defined medium for the growth of *B. subtilis* JH642 and its derivative GWB100. For growth experiments involving *B. methanolicus* MGA3, the chemically defined minimal medium MVcM was used (Brautaset et al., 2003). The composition of the MVcM medium is as follows: 23.3 mM K_2_HPO_4_, 10.8 mM NaH_2_PO_4_, 16 mM (NH_4_)_2_SO_4_; the pH of this medium is 7.2. 1-ml of a 1 000-fold concentrated trace metal solution (Brautaset et al., 2003) was added to 1 L of the basal MVcM medium and it was additionally supplemented with D-biotin (vitamin B_7_) (0.1 mg L^–1^), and vitamin B_12_ (0.01 mg L^–1^). Methanol was used as the carbon source for *B. methanolicus* MGA3 and was provided at a final concentration of 200 mM.

Growth of *B. subtilis* and *B. methanolicus* cultures was spectrophotometrically monitored at a wavelength of 578 nm (OD_578_). A *B. subtilis* single colony was picked from an LB agar plate and used to inoculate a 5 mL LB culture that was grown at 37°C to mid-exponential growth phase. Subsequently, 2 μL of this culture were used to inoculate a 20 mL culture (in SMM) (in a 100-mL Erlenmeyer flask) that was grown in a shaking water bath (at 37°C) overnight. From this type of pre-culture, all *B. subtilis* cultures were inoculated that were used for physiological experiments; typically to an OD_578_ of 0.1. Similarly, a single colony of *B. methanolicus* MGA3 was picked from SOB agar plates (incubated at 50°C) and used to inoculate 5 mL SOB rich medium (Hanahan, 1983) (in a 100 mL Erlenmeyer flasks); the cultures were grown at 50°C to mid-exponential growth phase (OD_578_ of about 0.5–1). From this culture, 15 μL were resuspended in 20 mL (in a 100 mL Erlenmeyer flasks) of MVcM medium with methanol as the carbon source; this culture was grown at 50°C in a shaking water bath overnight. From this type of pre-culture, all cultures used for physiological experiments with *B. methanolicus* were inoculated; typically to an OD_578_ of 0.1. For all growth experiments, the media were pre-warmed (to 37°C for *B. subtilis* and to 50°C for *B. methanolicus*) and the rotation of shaking water baths was set to 220 rpm.

The osmotic strength of the MVcM minimal medium was increased by addition of appropriate solutions from stocks of 5 M NaCl, 2 M KCl, 2 M lactose, or 2 M sucrose solutions to the final concentration indicated in the individual experiments. The osmolarity of these media was determined with a freezing point osmometer (Osmomat 3000, gonotec; Berlin, Germany). For osmoprotection growth assays (Boch et al., 1994), compatible solutes were added to the medium to a final concentration of 1 mM. When the use of L-glutamate as sole nitrogen and carbon sources by *B. subtilis* and *B. methanolicus* were assessed, the ammonium source [(NH_4_)_2_SO_4_] was replaced by 32 mM L-glutamate, and the carbon source (glucose for *B. subtilis* and methanol for *B. methanolicus*) was replaced by 40 mM L-glutamate.

### Preparation of Cell Extracts for ^13^C-NMR Spectroscopy

To evaluate the dominant organic osmolytes in the cytoplasm of *B. methanolicus* MGA3, ^13^C-NMR spectroscopy was used (Kuhlmann and Bremer, 2002; Bursy et al., 2007). For these experiments, the cells were grown in either MVcM or MVcM with 0.5 M NaCl until they reached either exponential or stationary growth phase. *B. methanolicus* MGA3 cultures that were not challenged by osmotic stress reached under these growth conditions an OD_578_ of 3.5, while those challenged with 0.5 M NaCl reached an OD_578_ of 1.5. Cells from four separately grown 500-ml cultures were combined to produce ethanolic extracts of soluble compounds. For these experiments, cells were harvested by centrifugation, and the cell pellet was extracted with 20 mL 80% (vol/vol) ethanol. After removing cellular debris by centrifugation, the supernatant was lyophilized. The dried material was then dissolved in 700 μL ^2^H_2_O supplemented with 1.2 mg of D_4_-3-(trimethylsilyl)propionate as an internal standard for ^13^C-NMR analytics. ^13^C-NMR spectra were recorded with a Bruker AC300 spectrometer operating at 75 MHz.

### HPLC Analysis of L-Glutamate

For quantitative HPLC analysis of L-glutamate, *B. methanolicus* MGA3 cells were cultivated in MVcM of different osmolarities until an OD_578_ of approximately 1 was reached. Cells were collected by centrifugation and the supernatant was separated from the pellet and stored at −20°C until further analysis. Cell pellets were evaporated to dryness, and the cell dry weight (CDW) was determined. Cell extracts of *B. methanolicus* MGA3 were prepared as previously described (Kuhlmann and Bremer, 2002). Amino acids present in the cell extracts and culture supernatants were derivatized with *o*-phtaldialdehyde (OPA) and fluorenylmethyloxycarbonyl chloride (FMOC-Cl) using an automated procedure based on a previously published method (Krömer et al., 2005). In brief, 0.5 μL of the reaction sample was mixed with 0.5 μL FMOC (1.25 mg mL^–1^ in acetonitrile) and incubated for 1 min at room temperature. Subsequently, 0.5 μL of 10 mg mL^–1^ OPA reagent dissolved in 1/75/75 (v/v/v) of 2-MCE/MeOH/borate (0.4 M; pH 10.2) was added. After 1 min incubation, the mixture was diluted with 36 μL H_2_O and injected into an HPLC system. The HPLC system (1260 Infinity, Agilent Technologies, Waldbronn, Germany) was equipped with a 150 × 4.6-mm Gemini 5 μm C18 110-Å column (Phenomenex, Aschaffenburg, Germany) and a fluorescence-detector (Agilent Technologies, Waldbronn, Germany). L-glutamate was detected at an excitation wavelength of 266 nm and at an emission wavelength of 305 nm. To allow an appropriate separation of amino acids, solvent A (40 mM phosphate buffer, pH 7.8) and solvent B (acetonitrile/methanol/water 45:45:10) solutions were used to create the following gradient: start, 0% B; 40.5 min, 40.5% B; 43 min, 61% B; 44 min, 82% B; 46.5 min, 100% B; and 47 min, 0% B. The flow rate was set to 1 mL min^–1^, and the temperature for the measurements was maintained at 40°C. L-glutamate was quantitated by using appropriate standards and the OpenLAB software suit (Agilent Technologies).

### Transport Assays

*B. methanolicus* and *B. subtilis* cultures were grown in the appropriated media to an OD_578_ of 0.5. To remove L-glutamate in the supernatant of *B. methanolicus* MGA3, the culture was washed with minimal media at isotonic osmolarity. Radiolabeled L-[U-^14^C]glutamate (specific activity 2.25 nCi nmol^–1^) was added to the cultures (final L-glutamate concentration in the transport assay was 1 mM; unlabeled L-glutamate was spiked with 0.14 μM radiolabeled L-[U-^14^C]glutamate) and uptake was monitored in 1-min time intervals by measuring the radioactivity accumulated by the cells in a Tri-Carb 2810 TR scintillation counter as described previously (Zaprasis et al., 2015).

### qPCR Analysis

For studying the expression of the L-glutamate biosynthetic genes (*gltA, gltA2*, and *yweB*) and of the *gltC* regulatory gene in response to the salinity of the growth medium, total RNA was extracted from *B. methanolicus* MGA3 cells using the peqGOLD TriFast Kit (VWR International GmbH, Erlangen, Germany). For these experiments, 10 mL of *B. methanolicus* MGA3 cultures (grown in MVcM with the appropriate NaCl concentration) were harvested in the early exponential phase (OD_578_ of 0.6) by centrifugation. The cells were re-suspended in peqGOLD TriFast reagent and disrupted with 0.1 mm glass beads using a Precellys 24 homogenizer (2 × 20 min 6500 rpm; VWR International GmbH, Erlangen, Germany). These samples were then further processed according to the instructions provided by the manufacturer of the peqGOLD TriFast Kit. To remove residual chromosomal DNA, the RNA containing solutions were treated with RNAse-free DNAse I (Life Technologies GmbH, Darmstadt, Germany). Quantitative PCR was run in a CFX96 PCR Detection System (Bio-Rad Laboratories GmbH, München, Germany), using the LightCycler RNA Master SYBR green I kit (Roche Diagnostics, Mannheim, Germany). The reaction was performed following the manufacturer’s instructions with denaturation at 95°C for 5 s, annealing at 58°C for 10 s, and elongation at 72°C for 10 s using 50 ng RNA and 0.5 μM of each primer. Data analysis was accomplished using the 2^–ΔΔ^ ^*CT*^ method (Livak and Schmittgen, 2001) and the amplification of 16S rRNA served as internal normalizers. The following DNA primers were used. *gltA*: 5′-GGACGTTATGGCGTTGGA-3′ and 5′-CGATATTTACAGGTACAGTTCGCC-3′; *gltA2*: 5′-CCGCCAG GAATATGATGCC-3′ and 5′-CCCCATCTCCTTCGCCA-3′, *gltC*: 5′-GTGGCCAAACGGGAACAC-3′ and 5′-GGGGTGAG CTTCACATTTCG-3′; yweB: 5′-CCAATACGCATGATGACAG-5′ and 5′-GGGAGGACAAACATGACG-3′; 16S rRNA: 5′-CTAC TGCTGCCTCCCGTAG-3′, 5′-AAGATGGCTTCGGCTATCAC-3′. *opuFA*: 5′-CGGATGCGGGAAACGAC-3′ and 5′-CTGC TGGAACATAGGCG-3′.

### Computer Analysis and Modeling of Protein Structures

The genome sequences of *B. methanolicus* MGA3 and of *B. methanolicus* PB1 (Heggeset et al., 2012) were retrieved from the IMG/M database^1^ (Nordberg et al., 2013); accession numbers CP007739 and Ga0248316, respectively. Amino acid sequences for the various Opu transporter protein from *B. subtilis* (Hoffmann and Bremer, 2017) and the OpuF system from *B. infantis* (Teichmann et al., 2018) were retrieved from the corresponding genome sequences and used to probe the presence of related genes in *B. methanolicus* strains MGA3 and PB1 by a BLASTP analysis The amino acid sequences of the *B. subtilis* enzymes for glycine betaine synthesis (GbsAB) (Boch et al., 1996) and osmostress adaptive production of the compatible solute L-proline (ProHJ) (Brill et al., 2011) were used to search for homologous sequences in the genomes of *B. methanolicus* MGA3 and *B. methanolicus* PB1. The amino acid sequences for ectoine biosynthetic enzymes (EctABC) were retrieved from the genome sequence of *Paenibacillus lautus* (Richter et al., 2019). Those for the synthesis of trehalose (OtsAB, TreYZ, TreS) were derived from the genome sequence of *C. glutamicum* (Wolf et al., 2003) and were used to search the *B. methanolicus* MGA3 and *B. methanolicus* PB1 genomes (Heggeset et al., 2012) for the presence of corresponding proteins.

The *in silico* structure of the substrate binding domain (OpuFBC) of the *B. methanolicus* MGA3 OpuF ABC transporter was derived by using the SWISS-MODEL server^2^ (Biasini et al., 2014). The crystal structure of the *Listeria monocytogenes* BilE substrate binding protein (PDB accession code: 4z7e) (Ruiz et al., 2016) was automatically chosen by the server as the most appropriate template to model the structure of the OpuFBC substrate binding protein domain; the overall model had a confidence score of 0.715 (Biasini et al., 2014). Graphical representations of *in silico* models of the substrate binding domain of the OpuF ABC choline transport system, BilE (Ruiz et al., 2016), and of the OpuBC substrate binding protein (Pittelkow et al., 2011) were prepared with the PyMol software package^3^ (Delano, 2002).

## Data Availability Statement

The original contributions presented in the study are included in the article/Supplementary Material, further inquiries can be directed to the corresponding author/s.

## Author Contributions

EB designed and supervised the study. CF performed all experiments. TH, OZ, and MF provided insights into the interpretation of the data. CF and EB wrote the manuscript with input from all other authors. All authors contributed to the article and approved the submitted version.

## Conflict of Interest

OZ and MF was employed by the company BASF SE. The remaining authors declare that the research was conducted in the absence of any commercial or financial relationships that could be construed as a potential conflict of interest. The authors declare that this study received funding from BASF SE. The funder was not involved in the study design, collection, analysis, interpretation of data, the writing of this article or the decision to submit it for publication.

## Footnotes

1 https://img.jgi.doe.gov/

2 https://swissmodel.expasy.org/

3 https://pymol.org/2/
