# Supplementary material for: Enhanced Glutamate Synthesis and Export by the Thermotolerant Emerging Industrial Workhorse Bacillus methanolicus in Response to High Osmolarity
Source: Front Microbiol. 2021 Apr 8;12:640980. doi: 10.3389/fmicb.2021.640980 (PMC8060640; doi:10.3389/fmicb.2021.640980)
Supplement: Supplementary file 1 [file Data_Sheet_1.PDF]

SUPPLEMENTAL MATERIAL for:

Enhanced glutamate synthesis and export by the thermotolerant emerging industrial workhorse *Bacillus methanolicus* in response to high osmolarity

Christine Frank[1], Tamara Hoffmann[1,2], Oskar Zelder[3], Max F. Felle[3], and Erhard Bremer[1,2,*]

[1]Laboratory for Microbiology, Department of Biology, Philipps-University Marburg, Marburg, Karl-von-Frisch Str. 8, D-35043 Marburg, Germany

[2]Center for Synthetic Microbiology (SYNMIKRO), Philipps-University Marburg, Hans-Meerwein Str. 6, D-35043 Marburg, Germany

[3]BASF SE, RBW/EC – A030, Carl-Bosch Str 38, D-67056 Ludwigshafen am Rhein, Germany

Running head: Osmostress response of *B. methanolicus*

***Correspondence to Dr. Erhard Bremer:** Laboratory for Microbiology, Department of Biology, Philipps-University Marburg, Karl-von-Frisch Strasse 8, D-35043 Marburg, Germany. Phone: (+49)-6421-2821529. Fax: (+49)-6421-2828979. E-Mail: bremer@staff.uni-marburg.de

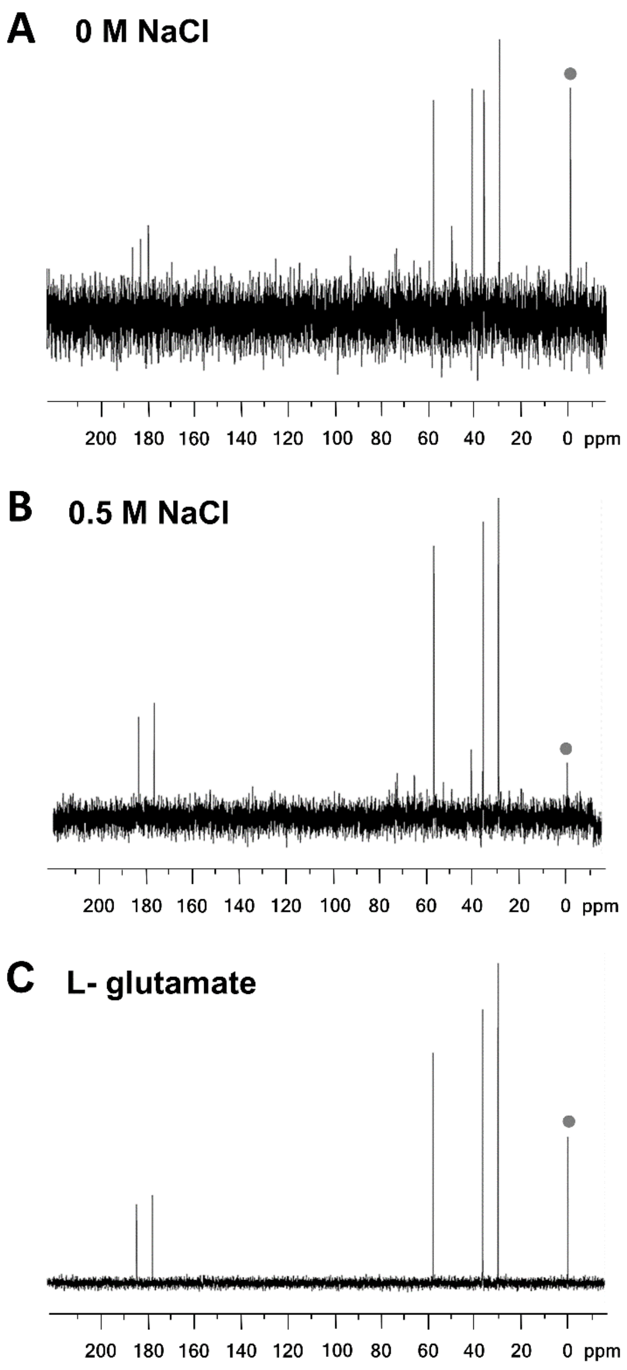

Supplementary Figure S1 ^{13}C NMR spectra of ethanolic *B. methanolicus* MGA3 cell extracts. Cultures of *B. methanolicus* MGA3 were grown at 50° C in MVcM in either (A) the absence, or (B) the presence of 0.5 M NaCl until they reached stationary phase. In the absence of salt stress, an OD_{578} of these cultures of about 3.5 was reached; in the presence of salt stress, an OD_{578} of these cultures of about 1.5 was reached. Ethanolic cell extracts were prepared and subjected to ^{13}C-NMR analysis on a Bruker AC300 spectrometer operating at 75 MHz. D_4-3-(trimethylsilyl)propionate was added to the extracts as an internal standard (its tracing is marked with a dot) and (C) the ^{13}C-NMR spectrum of L-glutamate (25 mg) was recorded as reference.

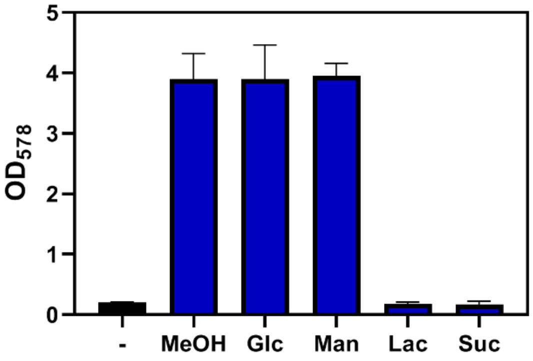

Supplementary Figure S2: Consumption of different C-sources by *B. methanolicus* MGA3. *B. methanolicus* MGA3 cells were grown in MVcM in the absence of a carbon source or in the presence of 200 mM methanol, 35 mM glucose (Glc), 50 mM mannitol (Man), 30 mM lactose (Lac), or 50 mM sucrose (Suc), respectively. The cultures were cultivated at 50° C in a shaking water bath and the growth yield was determined after 16 h of incubation by measuring the OD_{578}. The presented data were derived from two independently grown cultures.

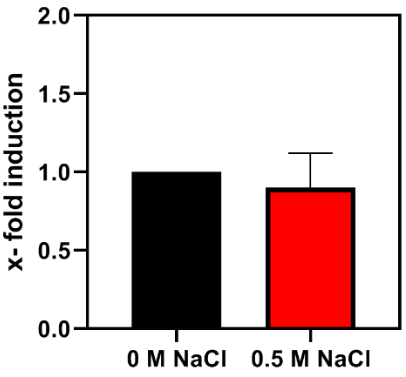

Supplementary Figure S3: qPCR analysis of the GDH encoding gene *yweB* of *B. methanolicus* MGA3. The qPCR data were derived from cultures of *B. methanolicus* MGA3 grown at 50° C in MVcM or MVcM with 0.5 M NaCl. The relative quantifications of the transcript levels are given. The value of the *yweB* transcripts detected in *B. methanolicus* MGA3 cells grown in MVcM 0.5 M NaCl was compared to the transcript level of cells cultivated in MVcM in the absence of additional NaCl; the cellular transcript level of *B. methanolicus* MGA3 grown in the absence of additional NaCl was set as one. The data were derived from RNA preparations of two independently grown *B. methanolicus* MGA3 cultures and each RNA preparation was measured three times. The RNA preparations used for the qPCR analysis of *yweB* were the same used for the qPCR analysis of the transcriptional pattern of the gltA, gltA2, and gltC genes, genes that showed a osmostress-responsive transcriptional profile (see main text).

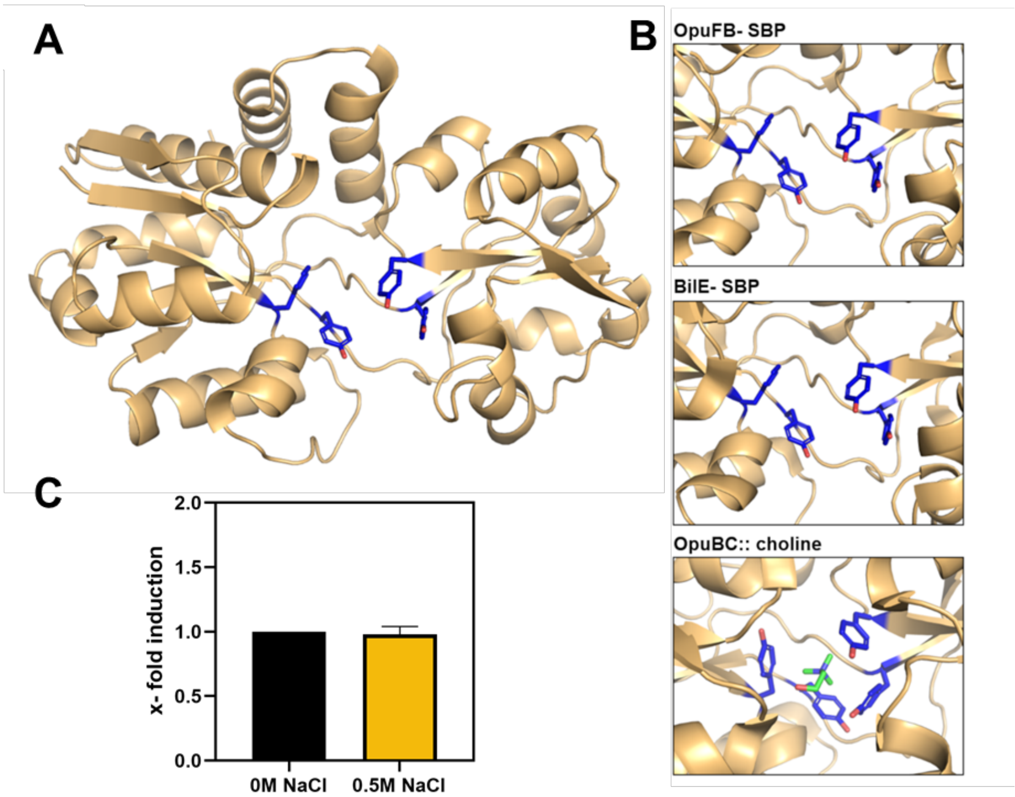

Supplementary Figure S4 *in silico* model of the OpuFBC substrate binding protein domain of the *B. methanolicus* MGA3 OpuF ABC transport system and qPCR analysis of its transcript by osmotically stressed cells. (A*) in silico* model of the overall structure of the *B. methanolicus* MGA3 substrate binding domain OpuFBC; it is based on the crystal structure of the BilE substrate binding protein from *Listeria monocytogenes* (PDB ID 4Z7E) (1). (B) A view into the substrate binding site of the actual crystal structure of BilE (1), of the modeled OpuFB protein (this study), and of the actual substrate binding protein (OpuBC) of the OpuB ABC transporter from *B. subtilis* in complex with choline (PDB ID: 3R6U) (2). Predicted and experimentally determined aromatic amino acids involved, in substrate binding are highlighted in blue. (C) qPCR analysis of the OpuFBC encoding genes *opuFBC* of *B. methanolicus* MGA3. The qPCR data were derived from cultures of *B. methanolicus* MGA3 grown at 50° C in MVcM or MVcM with 0.5 M NaCl. The relative quantification of the transcript level is given and the value of the *opuFBC* transcript detected in *B. methanolicus* MGA3 cells grown in the presence of 0.5 M NaCl was compared of the transcript level detected in *B. methanolicus* MGA3 cells grown in the absence of NaCl.

REFERENCES

1. Ruiz SJ, Schuurman-Wolters GK, Poolman B. 2016. Crystal structure of the substrate-binding domain from *Listeria monocytogenes* bile-resistance determinant BilE. *Crystals* 6:162.
2. Pittelkow M, Tschapek B, Smits SH, Schmitt L, Bremer E. 2011. The crystal structure of the substrate-binding protein OpuBC from *Bacillus subtilis* in complex with choline. *J. Mol. Biol.* 411:53-67.
